# Functional Interaction Between Caveolin 1 and LRRC8-Mediated Volume-Regulated Anion Channel

**DOI:** 10.3389/fphys.2021.691045

**Published:** 2021-10-01

**Authors:** Mikel Rezola, Aida Castellanos, Xavier Gasull, Núria Comes

**Affiliations:** ^1^Neurophysiology Laboratory, Physiology Unit, Department of Biomedicine, Medical School, Institute of Neurosciences, University of Barcelona, Barcelona, Spain; ^2^Institut d’Investigacions Biomèdiques August Pi i Sunyer (IDIBAPS), Barcelona, Spain

**Keywords:** volume-regulated anion channel, LRRC8A, caveolins, swelling-activated chloride current, protein interaction, channel surface localization

## Abstract

Volume-regulated anion channel (VRAC), constituted by leucine-rich repeat-containing 8 (LRRC8) heteromers, is crucial for volume homeostasis in vertebrate cells. This widely expressed channel has been associated with membrane potential modulation, proliferation, migration, apoptosis, and glutamate release. VRAC is activated by cell swelling and by low cytoplasmic ionic strength or intracellular guanosine 5′-*O*-(3-thiotriphosphate) (GTP-γS) in isotonic conditions. Despite the substantial number of studies that characterized the biophysical properties of VRAC, its mechanism of activation remains a mystery. Different evidence suggests a possible effect of caveolins in modulating VRAC activity: (1) Caveolin 1 (Cav1)-deficient cells display insignificant swelling-induced Cl^–^ currents mediated by VRAC, which can be restored by Cav1 expression; (2) Caveolin 3 (Cav3) knockout mice display reduced VRAC currents; and (3) Interaction between LRRC8A, the essential subunit for VRAC, and Cav3 has been found in transfected human embryonic kidney 293 (HEK 293) cells. In this study, we demonstrate a physical interaction between endogenous LRRC8A and Cav1 proteins, that is enhanced by hypotonic stimulation, suggesting that this will increase the availability of the channel to Cav1. In addition, LRRC8A targets plasma membrane regions outside caveolae of HEK 293 cells where it associates with non-caveolar Cav1. We propose that a rise in cell membrane tension by hypotonicity would flatten caveolae, as described previously, increasing the amount of Cav1 outside of caveolar structures interacting with VRAC. Besides, the expression of Cav1 in HEK Cav1- cells increases VRAC current density without changing the main biophysical properties of the channel. The present study provides further evidence on the relevance of Cav1 on the activation of endothelial VRAC through a functional molecular interaction.

## Introduction

The ability of eukaryotic cells to regulate their volume is crucial for key cellular processes including proliferation, migration, and apoptosis (Hoffmann et al., 2009; Chen et al., 2019). When vertebrate cells are challenged by changes in osmolarity conditions, they swell or shrink in order to restore their initial volume. Upon a hyposmotic shock, swollen cells can rapidly reduce (or restore) their volume by a mechanism known as regulatory volume decrease (Jentsch, 2016), which is commonly mediated by the coordinated action of ion channels that favor the efflux of K^+^, Cl^–^, taurine, and water (Okada and Maeno, 2001). Among these, the volume-regulated anion channel (VRAC) plays an essential role in the transport of Cl^–^ and small organic molecules to the extracellular space. VRAC currents are activated upon cell swelling in a voltage independent manner and show a characteristic inactivation at membrane potentials above +60 mV, modest outward rectification, and higher permeability to I^–^ and Br^–^ anions when compared to Cl^–^. Besides cell volume regulation, VRAC has been associated with the uptake of anticancer drugs and the release of signaling molecules such as glutamate, aspartate, and taurine (Jentsch et al., 2016).

In 2014, two research groups identified leucine-rich repeat-containing 8A (LRRC8A) as a required protein for the swelling-activated Cl^–^ currents mediated by VRAC (Qiu et al., 2014; Voss et al., 2014). LRRC8A was the first identified member of the leucine-rich repeat-containing 8 (LRRC8) family which contains four other members designated as LRRC8B–LRRC8E (Kubota et al., 2004). LRRC8 proteins form heteromeric channels that constitute the long-sought molecular identity of VRAC (Qiu et al., 2014; Voss et al., 2014). Several studies have revealed a hexameric assembly of LRRC8s (Deneka et al., 2018; Kasuya et al., 2018), which share a similar topology to pannexins (Abascal and Zardoya, 2012). Although the exact stoichiometry of VRAC is unknown, it has been proved that LRRC8A is indispensable to form functional channels together with at least one of the LRRC8B-LRRC8E subunits (Voss et al., 2014). Specific subunit composition of VRAC correlates with voltage dependence rectification, inactivation, permeability profile, and single-channel conductance (Qiu et al., 2014; Voss et al., 2014; Syeda et al., 2016). Each LRRC8 comprises a cytoplasmic C-terminal tail predicted to contain 17 leucine-rich repeat domains (LRRD) (Smits and Kajava, 2004) involved in protein interactions (Bella et al., 2008) and, as it has been hypothesized, important for gating mechanism (Gaitán-Peñas et al., 2016; Strange et al., 2019).

On the other hand, lipid rafts (LR) constitute a group of small (10–200 nm), dynamic, and transitory structures (Simons and Ikonen, 1997) highly enriched in sphingolipids and cholesterol (Schroeder et al., 1998). A specialized version of LR, characterized by the presence of the structural protein caveolin, is named caveolae. Caveolae are 50–80 nm Ω-shaped invaginations found on the plasma membrane of many vertebrates but specifically abundant in adipocytes, endothelia, fibroblasts, skeletal muscle cells, and myocytes. These structures have been related to functions as diverse as endocytosis, cell adhesion and migration, cholesterol homeostasis, mechanotransduction, and signal transduction (Parton and Simons, 2007). Caveolins comprise a family of oligomeric membrane proteins composed of caveolin 1 (Cav1), caveolin 2 (Cav2), and caveolin 3 (Cav3) (Williams and Lisanti, 2004). The first two are ubiquitous (Scherer et al., 1997), while Cav3 is the main structural protein of caveolae in muscle (Tang et al., 1996). Expression of Cav1 promotes caveolae formation in cells lacking caveolin (Fra et al., 1995), while its absence has been associated with caveolae disruption (Drab et al., 2001). In combination with PTRF-cavin proteins (Hill et al., 2008), Cav1 and Cav3 are needed for caveolae biogenesis in non-muscle and striated muscle cells, respectively (Parton et al., 2006). In contrast, Cav2 is not obligatory to form caveolae and seems to play a facilitator role for Cav1, contributing to caveolae formation (Parton and Simons, 2007).

Interestingly, caveolae can reversibly flatten in response to increases in volume-to-surface cell ratio in endothelial cells (Sinha et al., 2011). Disassembly of caveolae and the release of its plasma membrane to the bulk cell surface buffers membrane tension and prevents cell damage. Intriguingly, disruption of caveolae and re-distribution of Cav1 by cholesterol depletion (Westermann et al., 2005) facilitate VRAC currents (Klausen et al., 2006). In fact, transfection of caveolin 1 in Cav1-deficient cell lines restores the activity of VRAC (Trouet et al., 1999), and endothelial cells transfected with a mutated form of Cav1 show an impaired VRAC activity (Trouet et al., 2001). In this context, there is no further evidence on the functional association between Cav1 and VRAC. The study of the functional effects of the Cav1–LRRC8A interaction can shed some light on the mechanism behind the activation of VRAC currents.

## Materials and Methods

### Cell Culture, Transfections, and Treatments

Human embryonic kidney 293 (HEK 293), HEK Cav1-, and AD 293 cell lines were maintained in Dulbecco’s Modified Eagle’s Medium (DMEM), supplemented with 10% fetal bovine serum, 100 mg/ml L-glutamine, 100 U.I./ml penicillin, and 100 μg/ml streptomycin, and incubated in a humidified 5% CO_2_ atmosphere at 37°C. Once confluent, cells were passed with Trypsin-EDTA and were used in passages 3 to 7. Cell culture reagents were purchased from Sigma-Aldrich (Madrid, Spain).

Lentiviral infection was used to generate a HEK 293 cell clone where Cav1 is stably knockdown (HEK Cav1- cells) in the laboratory of Dr. Antonio Felipe (University of Barcelona). Briefly, Lv3p lentiviral particles codifying for the GFP protein and siRNA against Cav1 or scramble particles (Lv7p) were used (Pérez-Verdaguer et al., 2016). The expression of Cav1 in HEK Cav1- cells was periodically verified by western blot (WB) and cells were used up to 20 passages. HEK Cav1- cells were transfected with Cav1-YFP, Cav2-GFP, or YFP. Transfection was done with X-tremeGENE 9 DNA Transfection Reagent (Roche) when cells reached nearly 80% confluence. One μg of mCav1-YFP or hCav2-GFP plasmids was transfected into HEK Cav1- cells plated onto poly-lysine coated coverslips. Expression plasmids, mCav1-YFP (mouse caveolin-1α inserted in pEYFP-N1 between *Kpn*I and *Bam*HI) and hCav2-GFP (human caveolin-2β inserted in pEGFP-C1 between *Hin*dIII and *Apa*I), were provided by Dr. Antonio Felipe (University of Barcelona). Experiments were performed 24 h after transfection.

For osmotic challenges, confluent cells cultured in 10 cm dishes were washed 2× with phosphate-buffered saline (PBS) and incubated with 10 ml of isotonic or hypotonic solutions for 7 min at 37°C. The isotonic solution contained 115 mM NaCl, 5 mM 4-(2-hydroxyethyl)-1-piperazineethanesulfonic acid (HEPES), 11 mM glucose, and 65 mM saccharose; pH 7.3 with NaOH (300 mOsm/Kg). The hypotonic solution contained 115 mM NaCl, 5 mM HEPES, 11 mM glucose, and 5 mM saccharose; pH 7.3 with NaOH (255 mOsm/Kg, 15%). Osmolalities were measured with the use of a Vapro osmometer (Wescor, ELITechGroup, Puteaux, France), and carefully adjusted with sorbitol.

### Total Protein Extraction and Western Blot

To assay for protein levels, 24 h post-transfection cells were washed 2× with cold PBS of pH 7.4 and scraped with 1 ml of cold PBS of pH 7.4, containing aprotinin, leupeptin, pepstatin (1 μg/ml), and 1 mM phenylmethylsulfonyl fluoride (PMSF) as protease inhibitors (Sigma-Aldrich). After centrifugation, pellets were re-suspended with 0.5 ml of 1% Triton X-100 lysis buffer supplemented with the cocktail of protease inhibitors.

Total protein was quantified with the Pierce^TM^ BCA Protein Assay Kit (Thermo Scientific, Madrid, Spain). Polyvinylidene fluoride (PVDF) membranes were incubated with LRRC8A rabbit polyclonal antibody (1:500, Bethyl Laboratories, Montgomery, TX, United States), Cav1 rabbit monoclonal antibody (1:2,500, BD Biosciences, San Jose, CA, United States), clathrin mouse polyclonal antibody (1:5,000, Abcam, Cambridge, United Kingdom), Cav2 mouse monoclonal antibody (1:1,000, R&D Systems, Minneapolis, MN, United States), flotillin-1 mouse monoclonal antibody (1:1,000, BD Biosciences, San Jose, CA, United States), and β-actin mouse monoclonal antibody (1:5,000, Sigma-Aldrich). Since Cav1 is particularly abundant in HEK 293 cells, this protein may not be a suitable marker for lipid raft, thus we used flotillin-1 instead. Restore^TM^ PLUS Western blot stripping buffer (Thermo Scientific, Madrid, Spain) was used to strip off the PVDF membranes of the Cav1 antibody and reprove them with the Cav2 antibody. Secondary horseradish peroxidase (HRP)-conjugated goat anti-rabbit and goat anti-mouse antibodies were obtained from Jackson ImmunoResearch Laboratories (Ely, United Kingdom) and used at 1:20,000 to detect all proteins, except for β-actin for which it was used at 1:40,000. Immunoreactive bands were captured with the WesternBright^TM^ Quantum detection kit for HRP (Advansta, San Jose, CA, United States) and visualized on an LAS-3000 Mini gel Imaging System (Fujifilm, Minato, Tokyo, Japan). Experiments were done by triplicate.

### Co-immunoprecipitation

Protein extracts were obtained from HEK 293 cells with lysis buffer containing 1% Triton X-100 supplemented with the same protease inhibitors as above and the phosphatase inhibitor cocktail set II (Millipore, Burlington, MA, United States) (1:100). Subsequently, the extracts were homogenized mechanically and centrifuged at 16,000 rpms at 4°C for 30 min. Pellets were discarded, and protein from the supernatants was quantified using the Pierce^TM^ BCA Protein Assay kit (Thermo Scientific, Madrid, Spain). Initial protein extracts were prepared for WB. Meanwhile, 100–500 μg of protein was incubated with 2–4 μg of anti-Cav1 antibody, anti-LRRC8A antibody, or 0.4 μg of normal rabbit IgG (Santa Cruz Biotechnology, Dallas, TX, United States) at 4°C overnight. Later, 100 μl of protein A bound to sepharose beads (Sigma-Aldrich) was added in 500 μl of immunoprecipitated sample and allowed to incubate with stirring at 4°C for 2 h. The beads were centrifuged at 5,000 rpm for 5 min, and 100 μl of supernatants was collected. After washing with lysis buffer, samples with beads were prepared with 30 μl of loading buffer 2× with 10% β-mercaptoethanol and heated for 10 min at 75°C. Fifteen μl of supernatants were loaded on an electrophoresis gel for WB.

### Lipid Rafts Isolation

To isolate caveolae, HEK 293 cells were collected with a cell scrapper in PBS solution without Ca^2+^ or Mg^2+^, at 4°C. Next, cell suspensions were centrifuged at 1,000 rpms at 4°C for 10 min to concentrate the cells. Supernatants were discarded and the sedimented cells were lysed with 1 ml of MBSx1 MES (acid-morpholinoethanesulfonic) in PBS with 1% Triton X-100 supplemented with the protease inhibitors cocktail. Lysates were mechanically homogenized and extracts were mixed with 3 ml of a sucrose solution 53.28%, remaining at 40%. A continuous gradient was generated with 5 and 30% sucrose and tubes were adjusted and centrifuged at 39,000 rpms at 4°C for 21 h. Twelve fractions of 1 ml were collected from the surface of each tube and stored at −20°C.

### Electrophysiological Recordings

Patch clamp recordings were performed at 22–23°C using the whole-cell configuration without any leak subtraction. Electrodes were fabricated in a micropipette puller P-97 (Sutter Instruments, Novato, CA, United States) with a resistance of around 3 MΩ. An Ag/AgCl ground electrode mounted in a 3 M KCl agar bridge was employed. A conventional patch clamp rig was used, which consisted of an inverted microscope (Axiovert 35 M Zeiss, Oberkochen, Germany), an amplifier (Axopatch 200B), and pClamp 10 software suite from Molecular Devices (San Jose, CA, United States). Series resistance was kept at < 15 MΩ and compensated at 70–80%. Swelling-activated Cl^–^ currents were recorded using pulses from −80 to +80 mV in 20 mV voltage steps applied every 5 s from 0 mV. The intracellular pipette solution contained 150 mM NMDGCl, 1.2 mM MgCl_2_, 1.0 mM EGTA, 2.0 mM ATP, 0.5 mM GTP, and 10 mM HEPES; pH of 7.35 adjusted with tris-base (303 mOsm/Kg). The bath isotonic solution contained 150 mM NMDGCl, 0.5 mM MgCl_2_, 1.3 mM CaCl_2_, 10 mM HEPES, and 20 mM D-Sorbitol; pH of 7.35 adjusted with tris-base (298 ± 3 mOsm/Kg). The hyposmotic solution contained 100 mM NMDGCl, 0.5 mM MgCl_2_, 1.3 mM CaCl_2_, and 10 mM HEPES; pH of 7.35 adjusted with tris-base (210 ± 3 mOsm/Kg, 30%). Osmolalities were measured with an osmometer and adjusted with sorbitol. The current density was obtained by dividing the peak current amplitude at each voltage by the cell membrane capacitance (pA/pF). For activation curves, peak current amplitudes were normalized to the maximal current (I_max_). The mean of the data of each condition was plotted against used test potentials and fitted to Boltzmann function I = I_max_{1 + exp[(V_1/2_–V)/k]}^–1^, where V_1/2_ is the half-maximal activation voltage and *k* is the slope factor of the sigmoid curve.

### Data Analysis

All data are given as mean ± SEM. Statistical tests were performed with GraphPad Prism 9.0 software (GraphPad Software, San Diego, CA, United States) and statistical analysis was done with unpaired Student’s *t*-test. Entire I/V curves from HEK Cav1- versus transfected cells were analyzed with two-way ANOVA plus Bonferroni post-tests (^∗^*P* < 0.05, ^∗∗^*P* < 0.01, and ^∗∗∗^*P* < 0.001). A total of two patch clamp recordings were considered outliers using the ROUT test (GraphPad Prism 9.0 software, Q = 5%).

## Results

### Interaction of LRRC8A With Caveolin-1

**FIGURE 1:**
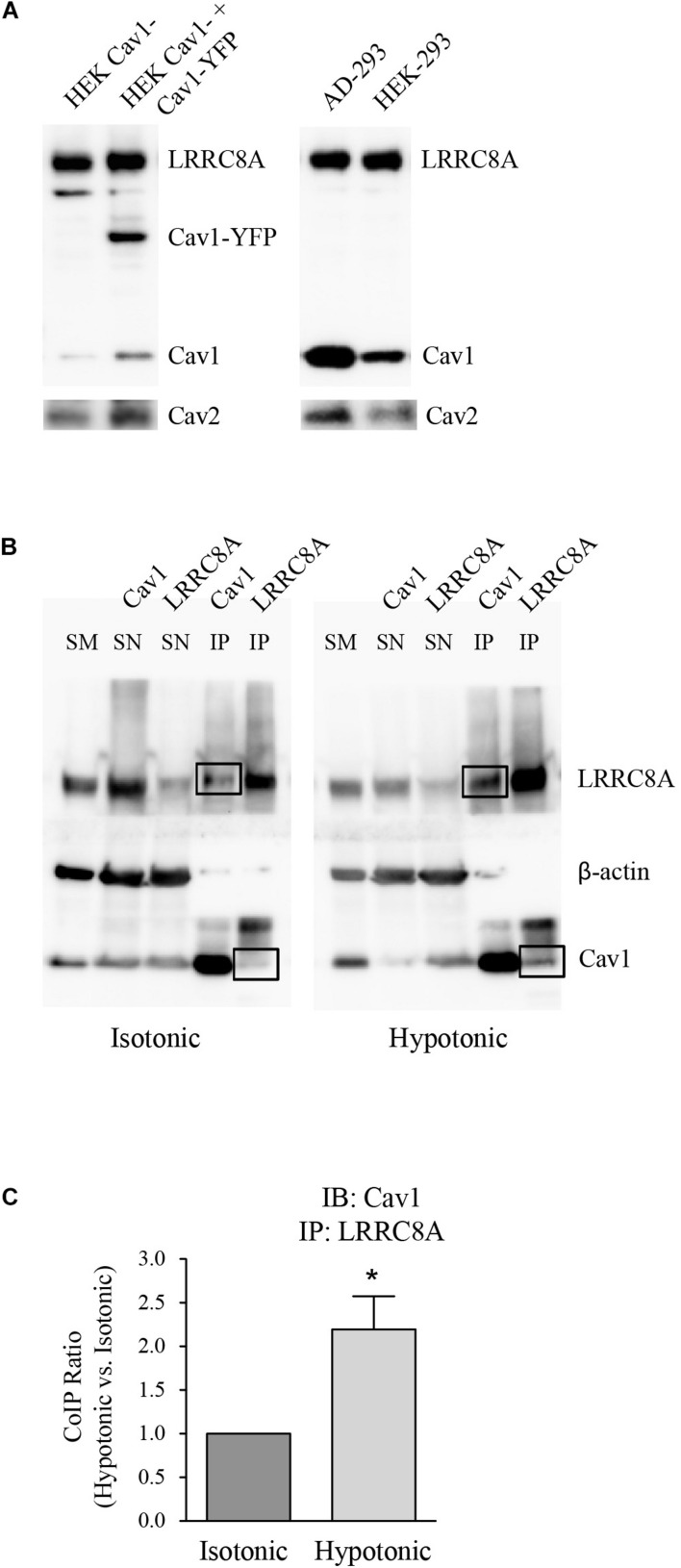
Expression and molecular association of LRRC8A and caveolin proteins. **(A)** Left: Representative western blot (WB) showing the endogenous expression of LRRC8A, Cav1, and Cav2 (after stripping off the Cav1 primary antibody), in non-transfected HEK Cav1- cells. Expression of these endogenous proteins in addition to Cav1-YFP was also determined in HEK Cav1- cells after transfection with Cav1-YFP. Right: Representative WB comparing endogenous LRRC8A, Cav1, and Cav2 (after stripping) in AD 293 and HEK 293 cell lines, both enriched with Cav1. **(B)** Representative blots of protein extracts subjected to immunoprecipitation. Endothelial HEK 293 cells exposed to either isotonic or hypotonic extracellular solutions were lysed and total protein extracts were immunoprecipitated using either Cav1 or LRRC8A-specific primary antibodies. Negative controls were done by the absence of antibodies and by immunoprecipitation with normal rabbit IgG, instead of specific primary antibodies (not shown). SM, starting material; SN, supernatant; IP, immunoprecipitate. The right panel shows how Cav1 protein co-immunoprecipitates to a greater extent under hypotonic conditions (black boxes). **(C)** Densitometry values for Cav1 immunoblot (IB) and LRRC8A IP quantification (LRRC8A/Cav1 ratio). Results are the mean data ± SEM of four independent experiments and the comparison between the isotonic and hypotonic groups is analyzed by unpaired *t*-tests (*p ≤ 0.05).

Leucine-rich repeat-containing 8A and Cav1 proteins were quantified in HEK 293 cells and HEK Cav1- cells (as a knock-down of Cav1 expression). Western blot analysis revealed that LRRC8A levels were not altered in HEK Cav1- cells after transfection with Cav1-YFP, in contrast to Cav1 which was significantly increased. To check the high abundance of Cav1 protein in HEK 293 cells, its expression was also determined in AD 293 cells, derived from the standard HEK 293 cell line but with improved cell adherence. In all the cell types that were tested, the expression of Cav2 changed according to Cav1, probably because they normally interact (Figure 1A). To study the interaction between endogenous LRRC8A and Cav1, we performed co-immunoprecipitation assays with HEK 293 cells exposed to isotonic or hypotonic conditions. In isotonicity, LRRC8A immunoprecipitated with Cav1, and interestingly, this interaction was significantly increased in hypotonic conditions known to activate VRAC (LRRC8A/Cav1 ratio = 2.20 ± 0.38 in hypotonic vs. isotonic, *n* = 4, *p* < 0.02; Figures 1B,C). On the contrary, we did not detect any interaction between LRRC8A and Cav2 proteins when LRRC8A was immunoprecipitated, whereas an expected Cav2 signal in the Cav1 immunoprecipitation was observed, confirming previous reports on Cav1–Cav2 interaction (not shown). In both osmotic conditions, reverse co-immunoprecipitation confirmed the physical interaction between LRRC8A and Cav1. The induction of this interaction by hypotonicity is further evidence of a functional effect of Cav1 on VRAC.

### Membrane Localization of LRRC8A in HEK 293 Cells

**FIGURE 2:**
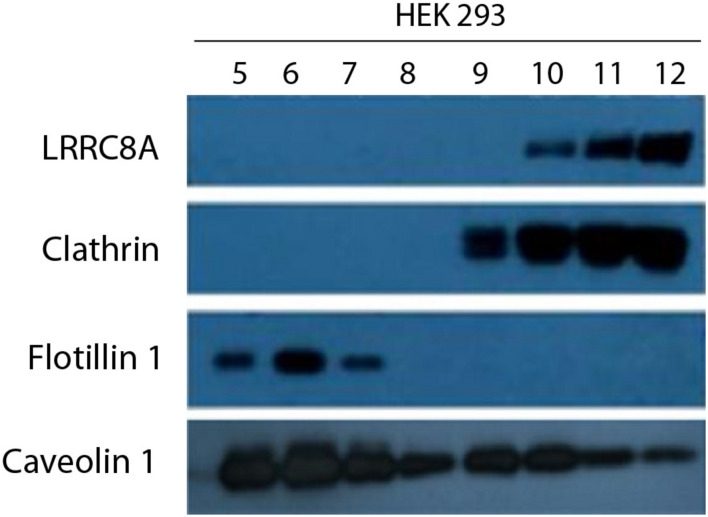
LRRC8A targeting to non-lipid raft domains in a caveolin-dependent manner. A representative experiment of a lipid raft isolation in HEK 293 cells. Twelve fractions from a sucrose-density gradient from total lysates were separated by SDS-PAGE and then immunoblotted with LRRC8A, Cav1, flotillin-1, or clathrin antibodies. Clathrin and flotillin-1 were used as a non-lipid raft and lipid raft markers, respectively. Notice that LRRC8A protein targeted Cav1 in non-floating fractions but not in floating fractions (enriched in lipid raft). Experiments were done in triplicate.

Several ion channels have been described to be spatially localized in lipid rafts in multiple cell lines (Martens et al., 2004; Maguy et al., 2006). As raft domains serve as scaffolding regions for cell signaling pathways, VRAC targeting lipid rafts may determine its regulation. To elucidate the specific membrane localization of VRAC in HEK 293 cells, we biochemically isolated lipid rafts as a non-dissolved membrane in non-ionic detergent. LRRC8A protein was found only in non-caveolar fractions enriched with clathrin (10–12) and not in caveolar ones enriched with flotillin-1 (5–7). This fact allowed us to state that VRAC is localized in regions of the plasma membrane with no caveolae (Figure 2). Clathrin and flotillin-1 have been extensively used in research as a non-lipid raft and lipid raft specific markers, respectively (Oliveras et al., 2014; Amsalem et al., 2018). It is interesting to note that free Cav1 can be detected also out of lipid rafts in endothelial cells as it has been found in other cell types expressing substantial amounts of Cav1 (Pérez-Verdaguer et al., 2018; Zabroski and Nugent, 2021). Since LRRC8A does not target lipid rafts in HEK 293 cells, we suggest that it may interact with the Cav1 located out of raft domains.

### Functional Effect of Cav1 on Volume-Regulated Anion Channel Activity in HEK Cav1- Cells

**FIGURE 3:**
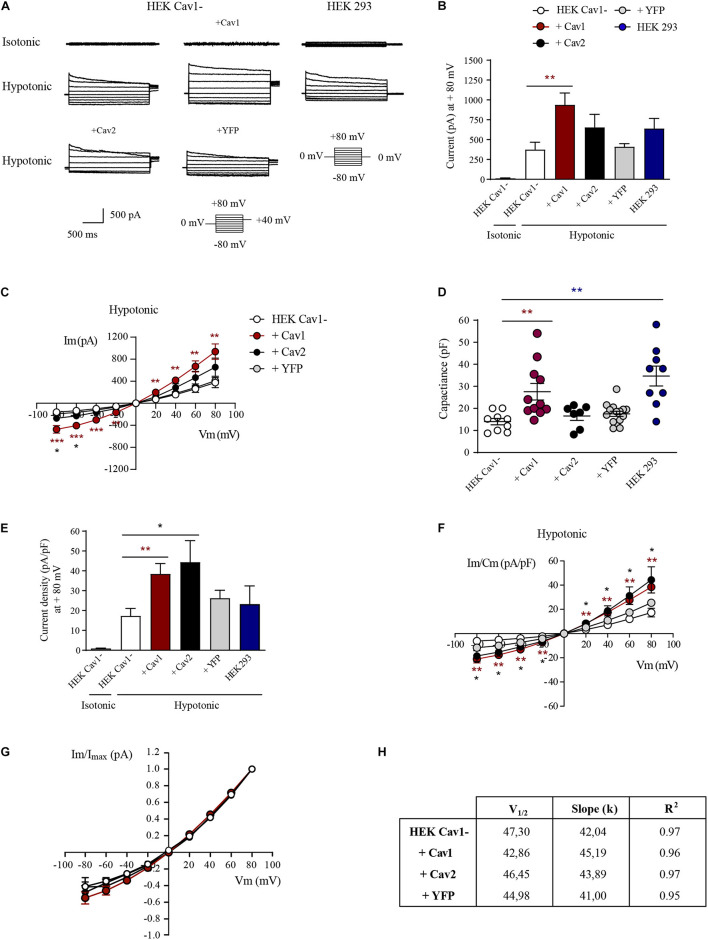
Caveolins modulate the current density of LRRC8-mediated VRAC. **(A)** Representative current traces elicited by 200 ms voltage sweeps ranging from –80 to +80 mV in 20 mV increments from a holding potential of 0 mV and a final tail pulse at +40 mV after each step (not shown). Current traces shown were recorded under isotonic conditions and after 3 min of exposure to the hypotonic bath solution. **(B)** Instantaneous current quantified at the peak of + 80 mV in HEK Cav1-, HEK Cav1- + Cav1, HEK Cav1- + Cav2, HEK Cav1- + YFP, and HEK 293 cells. All data are represented as means ± SEM, and comparisons between groups are analyzed by unpaired *t*-tests (*p ≤ 0.05, **p ≤ 0.005, and ***p ≤ 0.001). Comparisons between HEK Cav1- and HEK Cav1- + Cav1 are shown with asterisks. **(C)** Current-voltage relationship of instantaneous currents measured at the peak in each experimental group. **(D)** Membrane capacitance measured in pF as an indicator of cell size. Each dot represents a single recording in HEK Cav1- (white, *n* = 9), HEK Cav1- + Cav1 (dark red, *n* = 11), HEK Cav1- + Cav2 (black, *n* = 7), HEK Cav1- + YFP (gray, *n* = 10), and HEK 293 (blue, *n* = 9) cells. **(E)** Instantaneous current normalized by cell capacitance (current density) quantified at the peak of + 80 mV. **(F)** Current-voltage relationship of instantaneous normalized currents measured at the peak. HEK 293 cells (control) are not shown in I/V graphics to make the data clearer **(G)** Normalized currents represented to the maximum value (I/I_max_) versus each test potential fitted to Boltzmann function displayed a similar configuration in HEK Cav1- cells compared to HEK Cav1- cells transfected with Cav1, Cav2, or YFP. **(H)** Obtained V_1/2_ values did not significantly change in each condition in the same way as slope factors *k* of the sigmoid curve.

To decipher whether the physical interaction with Cav1 modulates the activity of VRAC, HEK Cav1- cells transfected with Cav1, Cav2, or YFP were used. In HEK Cav1- cells, resting currents at + 80 mV were minimal in isotonic conditions (13.55 ± 3.18 pA; *n* = 10) and VRAC currents rapidly developed after 3 min exposure to a hypotonic extracellular solution (374.71 ± 92.75 pA; *p* < 0.0001; Figures 3A–C). The characteristic time-dependent decay in the current activated at the most depolarized voltages can be observed in recordings of Figure 3A, while the VRAC outward current/voltage (I/V) relationship is shown in Figure 3C. Although in a few cells currents increased gradually reaching a maximum after 5 min of hypotonicity, current amplitude did not increase substantially after 3 min in most of the cells. In HEK Cav1- cells, transiently transfected with Cav1, hypotonic activation of VRAC was substantially enhanced at + 80 mV compared to HEK Cav1- cells (937.96 ± 141.90 pA; *n* = 10; *p* < 0.0001). VRAC activity was also increased upon hypotonicity after transfection with Cav2, but to a lesser extent than after Cav1 overexpression (654.40 ± 164.24 pA; *n* = 7; *p* < 0.0001), whereas transfection with YFP as negative control did not increase VRAC currents activated by hypotonicity compared to non-transfected HEK Cav1- cells (409.71 ± 40.01 pA; *n* = 15). Finally, HEK 293 cells displayed larger VRAC currents than HEK Cav1- cells, but significantly smaller than HEK Cav1- + Cav1 cells (639.62 ± 135.29 pA; *n* = 9; Figures 3A,B). Plasma membrane capacitance was measured from capacitative current transients after the establishment of whole cell mode in an isotonic condition. Capacitance values were significantly higher in HEK Cav1- cells transfected with Cav1 compared to non-transfected cells, suggesting the formation of invaginated caveolae by Cav1 overexpression (27.57 ± 3.73 pF vs. 16.02 ± 2.38 pF, respectively). In contrast, cell membrane capacitance was not modified when HEK Cav1- cells were transfected with Cav2 or YFP (Figure 3D). The current density was obtained by dividing the current amplitude for each cell by its capacitance value (pA/pF). Interestingly, a clear increase in current density was found at + 80 mV, not only in HEK Cav1- cells transfected with Cav1 (38.49 ± 4.94 pA/pF) but also in those transfected with Cav2 (44.39 ± 10.83 pA/pF), when compared to non-transfected cells (17.33 ± 3.57 pA/pF) and HEK 293 cells (23.25 ± 9.18 pA/pF, Figures 3E,F). Finally, normalized peak currents (I/I_max_) were plotted against every test potential and fitted to the Boltzmann function to explore the kinetics of voltage-dependent activation. Half-maximal activation voltages (V_1/2_) and slope factors (*k*) exhibited no significant differences between experimental groups, thereby showing that voltage dependence of activation was not altered by Cav1 and Cav2 that clearly modify the current density of VRAC (Figures 3G,H).

## Discussion

The biophysical properties of VRAC have been largely characterized, but specific molecular mechanisms governing its activation remain to be clarified. The classical procedure to activate currents mediated by VRAC in patch clamp experiments is to expose cells to an extracellular hypotonic solution that generates cell swelling. Nevertheless, understanding how the channel senses cell volume or the tension of the lipid bilayer remains a challenge. A notable reduction of VRAC currents in Cav1-deficient Caco-2, MCF-7, and T47D cell lines and its subsequent recovery by Cav1 transfection proved the pivotal role of Cav1 in channel activity (Trouet et al., 1999). Moreover, calf pulmonary artery endothelial cells transfected with the mutant Δ1–81, a dominant-negative Cav1, resulted in a deficient activation of VRAC (Trouet et al., 2001). Therefore, there is evidence that Cav1 might be required for VRAC activation. In this study, we describe that HEK Cav1- cells exhibit small swelling-activated Cl^–^ currents consistent with those mediated by VRAC, which is in agreement with that found in Cav1-deficient cell lines (severely or even totally impaired currents) (Trouet et al., 1999, 2001). Re-introduction of Cav1 recovers VRAC currents and, as expected, it produces a notable increase in the cell membrane, probably due to caveolae formation. Interestingly, regardless of the increase in membrane surface, a larger current density was observed (amount of current per membrane surface), suggesting that the interaction between Cav1 and VRAC may be functionally relevant. Accordingly, a similar effect on current density was detected when transfecting Cav2, although this did not alter the amount of cell membrane. Transfection of Cav1 or Cav2 likely produces an increase of caveolin outside caveolae, as these are easily saturated with Cav proteins (Pol et al., 2020).

The molecular mechanism through which caveolins could regulate the activity of VRAC is unknown. One possibility is through a direct association between the caveolin scaffolding domain of caveolins and the signature peptide enriched in aromatic residues, termed caveolin binding domain (CBD) (Couet et al., 1997). Interaction with caveolins has been proposed to have an inhibitory effect on a variety of signaling transduction molecules that include endothelial nitric oxide synthase and G protein-coupled receptors (Okamoto et al., 1998). We have analyzed the sequence of LRRC8A in search of a consensus CBD that is not present despite the protein containing numerous aromatic amino acids. In fact, a subsequent study showed that putative CBDs were not common amongst the caveolin interacting proteins and did not adopt a common structural orientation compatible for caveolin recognition (Collins et al., 2012). Alternatively, caveolins may facilitate assembly of ion channels to regulatory proteins or may control its sorting to membrane compartments, thereby increasing their availability to signaling proteins by an indirect mechanism. Little evidence exists in the literature about the modulation of VRAC by caveolins. Muscle-specific Cav3 knockout mice exhibited a reduction of VRAC current when compared to wild-type mice (Yamamoto et al., 2011). The association between LRRC8A and Cav3 transiently expressed in HEK 293 cells has been recently reported by co-immunoprecipitation and FRET (Turner et al., 2020). Besides direct or indirect regulation of VRAC by caveolins, its transfection increase the number of caveolae and, consequently, their ability to buffer volume changes. Caveolae seem to be involved in the regulation of mechanical tension in plasma membrane due to their invaginated structure (Sens and Turner, 2006) playing a mechanoprotective role in pathologies in which cells are subjected to high tension (Elliott et al., 2016). In fact, disassembly of caveolae and diffusion of Cav1 throughout the plasma membrane has been described after an increase in membrane tension (Tachikawa et al., 2017). For instance, mouse lung endothelial cells exposed to hypotonicity undergo a dissociation of Cav1–Cavin1 and a decrease of Cav1 from the caveolae surface (Sinha et al., 2011). Disruption of caveolar morphology removes membrane reserves, and it could accelerate the cell response to external hypotonicity. In this sense, in HEK Cav1- + Cav1 cells, VRAC should be less sensitive to cell swelling due to this buffer capacity of caveolae. Surprisingly, we found a higher VRAC current density, which suggests that Cav1 might promote the activation of the channel directly or indirectly and not through the ability of caveolae to buffer membrane changes.

On the other hand, loss of caveolae with methyl-β-cyclodextrin (MBCD) facilitates the activation of VRAC (Kozera et al., 2009; König et al., 2019). It has to be taken into account that MBCD produces depletion of membrane cholesterol and redistribution of caveolin from caveolae to non-buoyant fractions (Calaghan et al., 2008). Since caveolins modulate lateral segregation of cholesterol in the lipid bilayer (Meshulam et al., 2006), the amount of Cav1 at the cell surface might determine the sensitivity of VRAC to cell swelling through changes in biophysical properties of plasma membrane. In this line, Levitan and coworkers determined that cholesterol depletion in bovine endothelial cells resulted in VRAC enhancement while cholesterol enrichment suppressed its activity with no effect on the biophysical properties (Levitan et al., 2000). Likewise, cholesterol depletion increases the activity rate of VRAC in Ehrlich-Lettre ascites cells (Klausen et al., 2006). In this respect, a decreased number of caveolar structures by cholesterol depletion might promote a greater presence of Cav1 out of caveolae where VRAC is located in HEK 293 cells.

As VRAC could be relevant in endothelial mechanotransduction (Rosenthal et al., 2006), we explored its interaction with endogenous Cav1 in HEK 293 cells. Our data indicated that LRRC8A immunoprecipitates with Cav1 as previously reported in adipocytes (Zhang et al., 2017). Further, the physical interaction increases during hypotonic cell swelling, which could disperse Cav1 from caveolar structures to flat regions of the cell membrane. Some studies have found a low amount of free Cav at the plasma membrane at a steady state in different types of endothelial cells, which increased substantially under mechanical stress (Nassoy and Lamaze, 2012). Considering that Cav1 levels outside caveolae could be increased by membrane tension, VRAC interact with Cav1 out of these domains. LRRC8A has also been found in non-membrane microdomains and in membrane microdomains where VRAC is functionally associated with Na^+^/K^+^-ATPase in HT-29 cells (Fujii et al., 2018). Outside of caveolae, caveolins might be organized as oligomers or on lipid-rich platforms and associated with the distribution of lipids and proteins and regulation of actin cytoskeleton (Pol et al., 2020). It is important to highlight that caveolins outside caveolae are not only important in cells lacking caveolae. In fact, a number of proteins excluded from caveolae interact with non-caveolar caveolins or become available to caveolins when caveolae are disassembled (Shvets et al., 2015). Our results reveal that VRAC is located out of caveolae, where it might interact with Cav1 to modulate channel activation. A similar distribution has been found in endothelial-like trabecular meshwork cells that are also highly enriched with Cav1, and display endogenous VRAC activity (Gasull et al., 2019). Future studies are needed to identify the molecular determinants governing the interaction between Cav1 and LRRC8A proteins and the mechanisms underlying the regulation of LRRC8-mediated VRAC by caveolins.

## Data Availability Statement

The raw data supporting the conclusions of this article will be made available by the authors, without undue reservation.

## Author Contributions

AC, XG, and NC performed the electrophysiological recordings. AC and NC carried out the cellular cultures. MR performed the LRRC8A protein quantification and the co-immunoprecipitation assays. MR and NC were responsible for the lipid raft isolation. MR, AC, XG, and NC designed the experiments and did all the analysis and the statistics. NC supervised the study and wrote the text with the help of all the other authors. All authors critically read the manuscript and approved the final version.

## Conflict of Interest

The authors declare that the research was conducted in the absence of any commercial or financial relationships that could be construed as a potential conflict of interest.

## Publisher’s Note

All claims expressed in this article are solely those of the authors and do not necessarily represent those of their affiliated organizations, or those of the publisher, the editors and the reviewers. Any product that may be evaluated in this article, or claim that may be made by its manufacturer, is not guaranteed or endorsed by the publisher.

## References

[B1] Abascal F. Zardoya R. (2012). LRRC8 proteins share a common ancestor with pannexins, and may form hexameric channels involved in cell-cell communication. *BioEssays* 34 551–560. 10.1002/bies.201100173 22532330

[B2] Amsalem M. Poilbout C. Ferracci G. Delmas P. Padilla F. (2018). Membrane cholesterol depletion as a trigger of Nav1.9 channel-mediated inflammatory pain. *EMBO J.* 37 1–19. 10.15252/embj.201797349 29459435 PMC5897772

[B3] Bella J. Hindle K. L. McEwan P. A. Lovell S. C. (2008). The leucine-rich repeat structure. *Cell. Mol. Life Sci.* 65 2307–2333. 10.1007/s00018-008-8019-0 18408889 PMC11131621

[B4] Calaghan S. Kozera L. White E. (2008). Compartmentalisation of cAMP-dependent signalling by caveolae in the adult cardiac myocyte. *J. Mol. Cell. Cardiol.* 45 88–92. 10.1016/j.yjmcc.2008.04.004 18514221

[B5] Chen L. König B. Liu T. Pervaiz S. Razzaque Y. S. Stauber T. (2019). More than just a pressure release valve: physiological roles of volume-regulated LRRC8 anion channels. *Biol. Chem.* 400 1481–1496. 10.1515/hsz-2019-0189 31091194

[B6] Collins B. M. Davis M. J. Hancock J. F. Parton R. G. (2012). Structure-based reassessment of the caveolin signaling model: do caveolae regulate signaling through caveolin-protein interactions? *Dev. Cell* 23 11–20. 10.1016/j.devcel.2012.06.012 22814599 PMC3427029

[B7] Couet J. Li S. Okamoto T. Ikezu T. Lisanti M. (1997). Identification of peptide and protein ligands for the caveolin-scaffolding domain. Implications for the interaction of caveolin with caveolae-associated proteins. *J. Biol. Chem.* 272 6525–6533. 9045678 10.1074/jbc.272.10.6525

[B8] Deneka D. Sawicka M. Lam A. K. M. Paulino C. Dutzler R. (2018). Structure of a volume-regulated anion channel of the LRRC8 family. *Nature* 558 254–259. 10.1038/s41586-018-0134-y 29769723

[B9] Drab M. Verkade P. Elger M. Kasper M. Lohn M. Lauterbach B. (2001). Loss of caveolae, vascular dysfunction, and pulmonary defects in Caveolin-1 gene-disrupted mice. *Science (80)* 293:242452. 10.1126/science.1062688 11498544

[B10] Elliott M. H. Ashpole N. E. Gu X. Herrnberger L. McClellan M. E. Griffith G. L. (2016). Caveolin-1 modulates intraocular pressure: implications for caveolae mechanoprotection in glaucoma. *Sci. Rep.* 6:37127. 10.1038/srep37127 27841369 PMC5107904

[B11] Fra A. M. Williamson E. Simons K. Parton R. G. (1995). De novo formation of caveolae in lymphocytes by expression of VIP21-caveolin. *Proc. Natl. Acad. Sci. U.S.A.* 92 8655–8659. 10.1073/pnas.92.19.8655 7567992 PMC41025

[B12] Fujii T. Shimizu T. Yamamoto S. Funayama K. Fujita K. Tabuchi Y. (2018). Crosstalk between Na+,K+-ATPase and a volume-regulated anion channel in membrane microdomains of human cancer cells. *Biochim. Biophys. Acta - Mol. Basis Dis.* 1864 3792–3804. 10.1016/j.bbadis.2018.09.014 30251696

[B13] Gaitán-Peñas H. Gradogna A. Laparra-Cuervo L. Solsona C. Fernández-Dueñas V. Barrallo-Gimeno A. (2016). Investigation of LRRC8-Mediated volume-regulated anion currents in xenopus oocytes. *Biophys. J.* 111 1429–1443. 10.1016/j.bpj.2016.08.030 27705766 PMC5052465

[B14] Gasull X. Castany M. Castellanos A. Rezola M. Andrés-Bilbé A. Canut M. I. (2019). The LRRC8-mediated volume-regulated anion channel is altered in glaucoma. *Sci. Rep.* 9 1–16. 10.1038/s41598-019-41524-3 30931966 PMC6443673

[B15] Hill M. M. Bastiani M. Luetterforst R. Kirkham M. Kirkham A. Nixon S. J. (2008). PTRF-Cavin, a conserved cytoplasmic protein required for caveola formation and function. *Cell* 132 113–124. 10.1016/j.cell.2007.11.042 18191225 PMC2265257

[B16] Hoffmann E. K. Lambert I. H. Pedersen S. F. (2009). Physiology of cell volume regulation in vertebrates. *Physiol. Rev.* 89 193–277. 10.1152/physrev.00037.2007 19126758

[B17] Jentsch T. J. (2016). VRACs and other ion channels and transporters in the regulation of cell volume and beyond. *Nat. Rev. Mol. Cell Biol.* 17 293–307. 10.1038/nrm.2016.29 27033257

[B18] Jentsch T. J. Lutter D. Planells-Cases R. Ullrich F. Voss F. K. (2016). VRAC: molecular identification as LRRC8 heteromers with differential functions. *Pflugers Arch. Eur. J. Physiol.* 468 385–393. 10.1007/s00424-015-1766-5 26635246

[B19] Kasuya G. Nakane T. Yokoyama T. Jia Y. Inoue M. Watanabe K. (2018). Cryo-EM structures of the human volume-regulated anion channel LRRC8. *Nat. Struct. Mol. Biol.* 25 797–804. 10.1038/s41594-018-0109-6 30127360

[B20] Klausen T. K. Hougaard C. Hoffmann E. K. Pedersen S. F. (2006). Cholesterol modulates the volume-regulated anion current in Ehrlich-Lettre ascites cells via effects on Rho and F-actin. *Am. J. Physiol. - Cell Physiol.* 291 757–771. 10.1152/ajpcell.00029.2006 16687471

[B21] König B. Hao Y. Schwartz S. Plested A. J. R. Stauber T. (2019). A FRET sensor of C-terminal movement reveals VRAC activation by plasma membrane DAG signaling rather than ionic strength. *Elife* 8 1–20. 10.7554/eLife.45421.001 PMC6597245 31210638

[B22] Kozera L. White E. Calaghan S. (2009). Caveolae act as membrane reserves which limit mechanosensitive ICl, swell channel activation during swelling in the rat ventricular myocyte. *PLoS One* 4:e8312. 10.1371/journal.pone.0008312 20011535 PMC2788708

[B23] Kubota K. Kim J. Y. Sawada A. Tokimasa S. Fujisaki H. Matsuda-hashii Y. (2004). LRRC8 involved in B cell development belongs to a novel family of leucine-rich repeat proteins. *FEBS Lett.* 564 147–152. 10.1016/S0014-5793(04)00332-1 15094057

[B24] Levitan I. Christian A. E. Tulenko T. N. Rothblat G. H. (2000). Membrane cholesterol content modulates activation of volume-regulated anion current in bovine endothelial cells. *J. Gen. Physiol.* 115 405–416. 10736308 10.1085/jgp.115.4.405 PMC2233759

[B25] Maguy A. Hebert T. E. Nattel S. (2006). Involvement of lipid rafts and caveolae in cardiac ion channel function. *Cardiovasc. Res.* 69 798–807. 10.1016/j.cardiores.2005.11.013 16405931

[B26] Martens J. R. O’Connell K. Tamkun M. (2004). Targeting of ion channels to membrane microdomains: localization of K V channels to lipid rafts. *Trends Pharmacol. Sci.* 25 16–21. 10.1016/j.tips.2003.11.007 14723974

[B27] Meshulam T. Simard J. R. Wharton J. Hamilton J. A. Pilch P. F. (2006). Role of caveolin-1 and cholesterol in transmembrane fatty acid movement. *Biochemistry* 45 2882–2893. 10.1021/bi051999b 16503643

[B28] Nassoy P. Lamaze C. (2012). Stressing caveolae new role in cell mechanics. *Trends Cell Biol.* 22 381–389. 10.1016/j.tcb.2012.04.007 22613354

[B29] Okada Y. Maeno E. (2001). Apoptosis, cell volume regulation and volume-regulatory chloride channels. *Comp. Biochem. Physiol. A. Mol. Integr. Physiol.* 130 377–383. 11913451 10.1016/s1095-6433(01)00424-x

[B30] Okamoto T. Schlegel A. Scherer P. E. Lisanti M. P. (1998). Caveolins, a family of scaffolding proteins for organizing “preassembled signaling complexes” at the plasma membrane. *J. Biol. Chem.* 273 5419–5422. 10.1074/jbc.273.10.5419 9488658

[B31] Oliveras A. Roura-Ferrer M. Solé L. De La Cruz A. Prieto A. Etxebarria A. (2014). Functional assembly of Kv7.1/Kv7.5 channels with emerging properties on vascular muscle physiology. *Arterioscler. Thromb. Vasc. Biol.* 34 1522–1530. 10.1161/ATVBAHA.114.303801 24855057

[B32] Parton R. G. Hanzal-Bayer M. Hancock J. F. (2006). Biogenesis of caveolae: a structural model for caveolin-induced domain formation. *J. Cell Sci.* 119 787–796. 10.1242/jcs.02853 16495479

[B33] Parton R. G. Simons K. (2007). The multiple faces of caveolae. *Nat. Rev. Mol. Cell Biol.* 8 185–194. 10.1038/nrm2122 17318224

[B34] Pérez-Verdaguer M. Capera J. Martínez-Mármol R. Camps M. Comes N. Tamkun M. M. (2016). Caveolin interaction governs Kv1.3 lipid raft targeting. *Sci. Rep.* 6:22453. 10.1038/srep22453 26931497 PMC4773814

[B35] Pérez-Verdaguer M. Capera J. Ortego-Domínguez M. Bielanska J. Comes N. Montoro R. J. (2018). Caveolar targeting links Kv1.3 with the insulin-dependent adipocyte physiology. *Cell. Mol. Life Sci.* 75 4059–4075. 10.1007/s00018-018-2851-7 29947924 PMC11105548

[B36] Pol A. Morales-Paytuví F. Bosch M. Parton R. G. (2020). Non-caveolar caveolins - Duties outside the caves. *J. Cell Sci.* 133:jcs241562. 10.1242/jcs.241562 32393675

[B37] Qiu Z. Dubin A. E. Mathur J. Tu B. Reddy K. Miraglia L. J. (2014). SWELL1, a plasma membrane protein, is an essential component of volume-regulated anion channel. *Cell* 157 447–458. 10.1016/j.cell.2014.03.024 24725410 PMC4023864

[B38] Rosenthal R. Bakall B. Kinnick T. Peachey N. Wimmers S. Wadelius C. (2006). Expression of bestrophin-1, the product of the VMD2 gene, modulates voltage-dependent Ca2+ channels in retinal pigment epithelial cells. *FASEB J.* 20 178–180. 10.1096/fj.05-4495fje 16282372

[B39] Scherer P. E. Lewis Y. Volonte D. Engelman J. A. Galbiati F. Couet J. (1997). Cell-type and tissue-specific expression of Caveolin-2. *J. Biol. Chem.* 272 29337–29346. 9361015 10.1074/jbc.272.46.29337

[B40] Schroeder R. J. Ahmed S. N. Zhu Y. London E. Brown D. A. (1998). Cholesterol and sphingolipid enhance the Triton X-100 insolubility of glycosylphosphatidylinositol-anchored proteins by promoting the formation of detergent-insoluble ordered membrane domains. *J. Biol. Chem.* 273 1150–1157. 10.1074/jbc.273.2.1150 9422781

[B41] Sens P. Turner M. S. (2006). Budded membrane microdomains as tension regulators. *Phys. Rev. E - Stat. Nonlinear, Soft Matt. Phys.* 73 1–4. 10.1103/PhysRevE.73.031918 16605569

[B42] Shvets E. Bitsikas V. Howard G. Hansen C. G. Nichols B. J. (2015). Dynamic caveolae exclude bulk membrane proteins and are required for sorting of excess glycosphingolipids. *Nat. Commun.* 6 1–16. 10.1038/ncomms7867 25897946 PMC4410672

[B43] Simons K. Ikonen E. (1997). Functional rafts in cell membranes. *Nature* 387 569–572. 10.1038/42408 9177342

[B44] Sinha B. Köster D. Ruez R. Gonnord P. Bastiani M. Abankwa D. (2011). Cells respond to mechanical stress by rapid disassembly of caveolae. *Cell* 144 402–413. 10.1016/j.cell.2010.12.031 21295700 PMC3042189

[B45] Smits G. Kajava A. V. (2004). LRRC8 extracellular domain is composed of 17 leucine-rich repeats. *Mol. Immunol.* 41 561–562. 10.1016/j.molimm.2004.04.001 15183935

[B46] Strange K. Yamada T. Denton J. S. (2019). A 30-year journey from volume-regulated anion currents to molecular structure of the LRRC8 channel. *J. Gen. Physiol.* 151 100–117. 10.1085/jgp.201812138 30651298 PMC6363415

[B47] Syeda R. Qiu Z. Dubin A. E. Murthy S. E. Florendo M. N. Mason D. E. (2016). LRRC8 proteins form volume-regulated anion channels that sense ionic strength. *Cell* 164 499–511. 10.1016/j.cell.2015.12.031 26824658 PMC4733249

[B48] Tachikawa M. Morone N. Senju Y. Sugiura T. Hanawa-Suetsugu K. Mochizuki A. (2017). Measurement of caveolin-1 densities in the cell membrane for quantification of caveolar deformation after exposure to hypotonic membrane tension. *Sci. Rep.* 7:7794. 10.1038/s41598-017-08259-5 28798329 PMC5552771

[B49] Tang Z. Scherer P. E. Okamoto T. Song K. Chu C. Kohtz D. S. (1996). Molecular cloning of caveolin-3, a novel member of the caveolin gene family expressed predominantly in muscle. *J. Biol. Chem.* 271 2255–2261. 10.1074/jbc.271.4.2255 8567687

[B50] Trouet D. Hermans D. Droogmans G. Nilius B. Eggermont J. (2001). Inhibition of volume-regulated anion channels by dominant-negative caveolin-1. *Biochem. Biophys. Res. Commun.* 284 461–465. 11394902 10.1006/bbrc.2001.4995

[B51] Trouet D. Nilius B. Jacobs A. Remacle C. Droogmans G. Eggermont J. (1999). Caveolin-1 modulates the activity of the volume-regulated chloride channel. *J. Physiol.* 520 113–119. 10517805 10.1111/j.1469-7793.1999.t01-1-00113.x PMC2269555

[B52] Turner D. G. Tyan L. Stroebel S. Deguire F. Lang D. Glukhov A. (2020). Caveolin-3 enriches and dynamically interacts with Swell1. *Circ. Res.* 127:A423.

[B53] Voss F. K. Ullrich F. Münch J. Lazarow K. Lutter D. Mah N. (2014). Identification of LRRC8 heteromers as an essential component of the volume-regulated anion channel VRAC. *Science* 344 634–638. 10.1126/science.1252826 24790029

[B54] Westermann M. Steiniger F. Richter W. (2005). Belt-like localisation of caveolin in deep caveolae and its re-distribution after cholesterol depletion. *Histochem. Cell Biol.* 123 613–620. 10.1007/s00418-004-0750-5 15889267

[B55] Williams T. M. Lisanti M. P. (2004). The caveolin proteins. *Genome Biol.* 5 1–8. 10.1186/gb-2004-5-3-214 15003112 PMC395759

[B56] Yamamoto S. Kita S. Iyoda T. Yamada T. Iwamoto T. (2011). Caveolin-3 regulates the volume-regulated anion channel in mouse ven- tricular cells. *Biophys. J.* 100:267A.

[B57] Zabroski I. O. Nugent M. A. (2021). Lipid raft association stabilizes VEGF receptor 2 in endothelial cells. *Int. J. Mol. Sci.* 22 1–15. 10.3390/ijms22020798 33466887 PMC7830256

[B58] Zhang Y. Xie L. Gunasekar S. K. Tong D. Mishra A. Gibson W. J. (2017). SWELL1 is a regulator of adipocyte size, insulin signalling and glucose homeostasis. *Nat. Cell Biol.* 19 504–517. 10.1038/ncb3514 28436964 PMC5415409

